# Supplementary material for: TLR7: A Key Prognostic Biomarker and Immunotherapeutic Target in Lung Adenocarcinoma
Source: Biomedicines. 2025 Jan 9;13(1):151. doi: 10.3390/biomedicines13010151 (PMC11761590; doi:10.3390/biomedicines13010151)
Supplement: Supplementary file 1 [file biomedicines-13-00151-s001.zip › Supplementary file S1.pdf]

Supplementary Figures

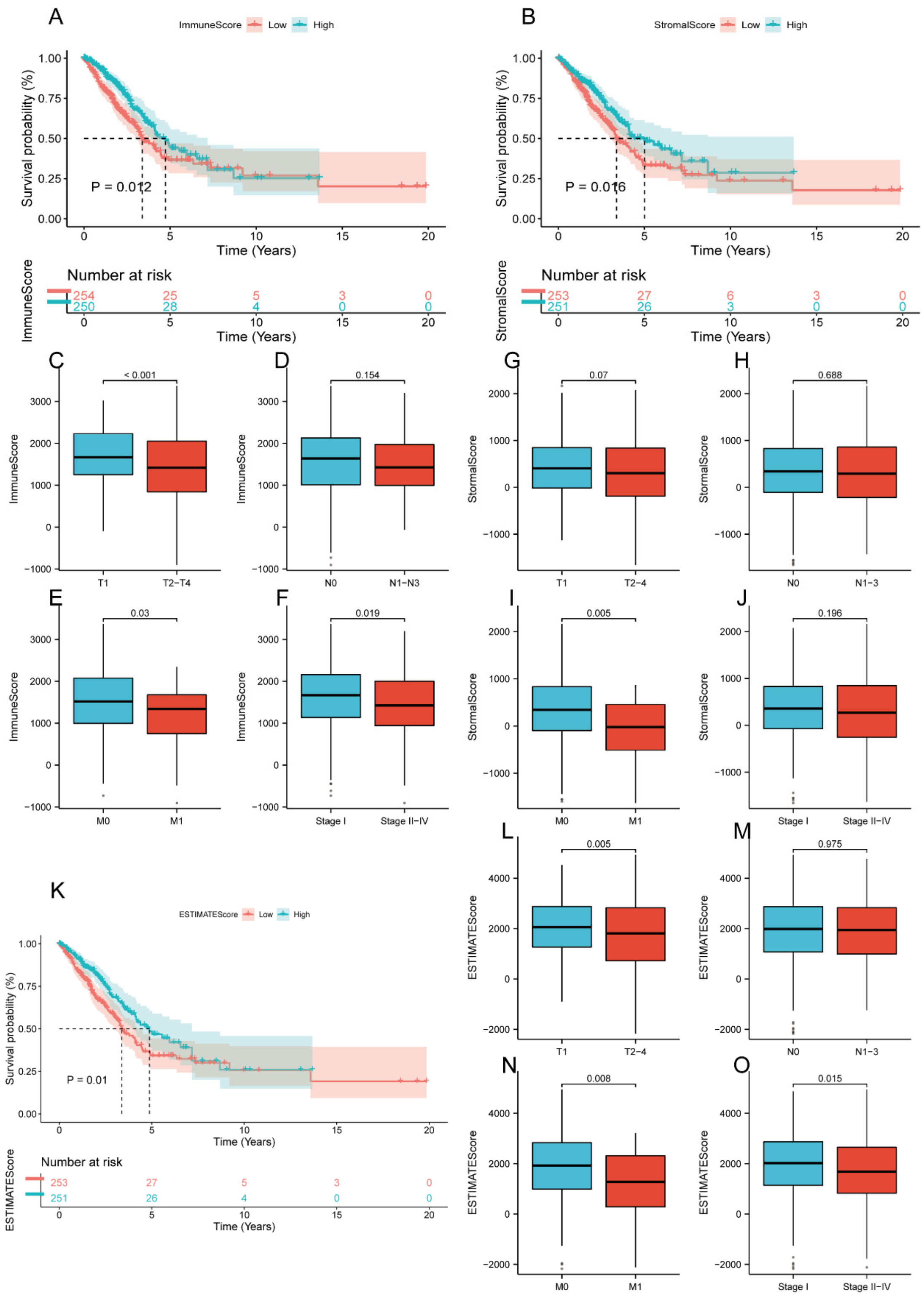

Supplementary Figure S1. Correlation of scores with survival and clinicopathologic staging characteristics of LUAD patients. (**A**) Kaplan–Meier survival analysis of LUAD patients determined by comparing the median to classify the ImmuneScore as high or low, P = 0.012 by log-rank test. (**B**) Kaplan–Meier survival curves for StromalScore, p = 0.016 by log-rank test. (**C–F**) Distribution of ImmuneScoresin clinical TNM and stage by Kruskal–Wallis rank sum test, p < 0.001, p = 0.154, p = 0.03, and p = 0.019, respectively. (**G–J**) Distribution of StormalScores in clinical TNM and stage by Kruskal–Wallis rank sum test, p= 0.07, p = 0.688, p = 0.005 and p = 0.196, respectively. (**K**) Survival analysis of LUAD patients grouped by ESTIMATEScore using the Kaplan–Meier method (log-rank test, p = 0.01). (**L–O**) Distribution of ESTIMATEScore in clinical TNM and Stage by Kruskal–Wallis rank sum test. p = 0.005, p = 0.975, p = 0.008 and p = 0.015, respectively.

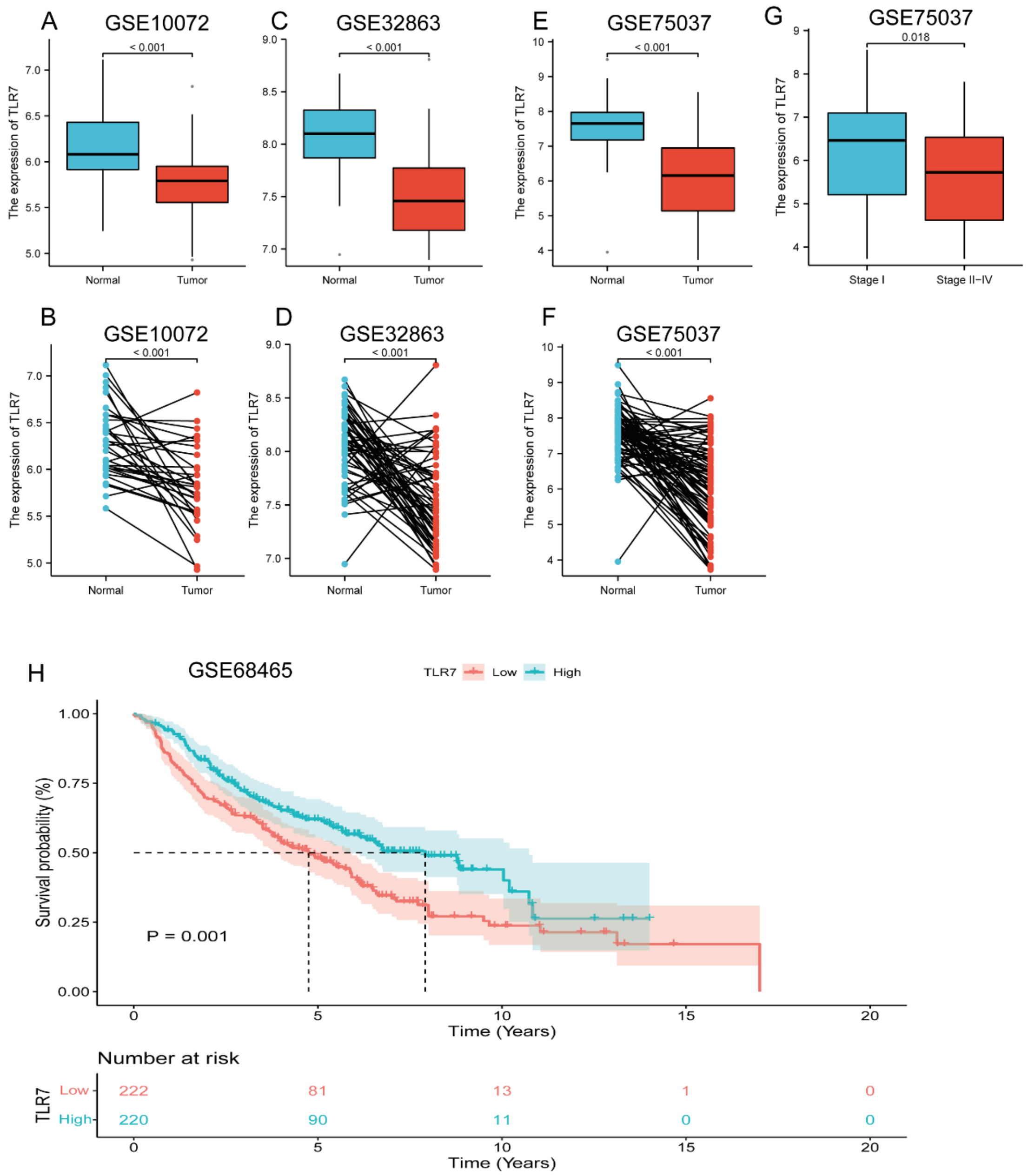

Supplementary Figure S2. Correlation of TLR7 expression with survival and clinical characteristics. (**A,C,E**) TCGA data and three GEO datasets analyzed in all normal and tumor samples, $p < 0.001$. (**B,D,F**) TCGA data and three GEO datasets were analyzed in paired normal and tumor samples from the same patient for TLR7 expression, $p < 0.001$. (**G**) Relationship between TLR7 expression and clinical early and late-Stage grading in GSE75037 Wilcoxon rank sum test was used as a statistical significance test, p=0.018. (**H**) Survival analysis of LUAD patients with different TLR7 expression.

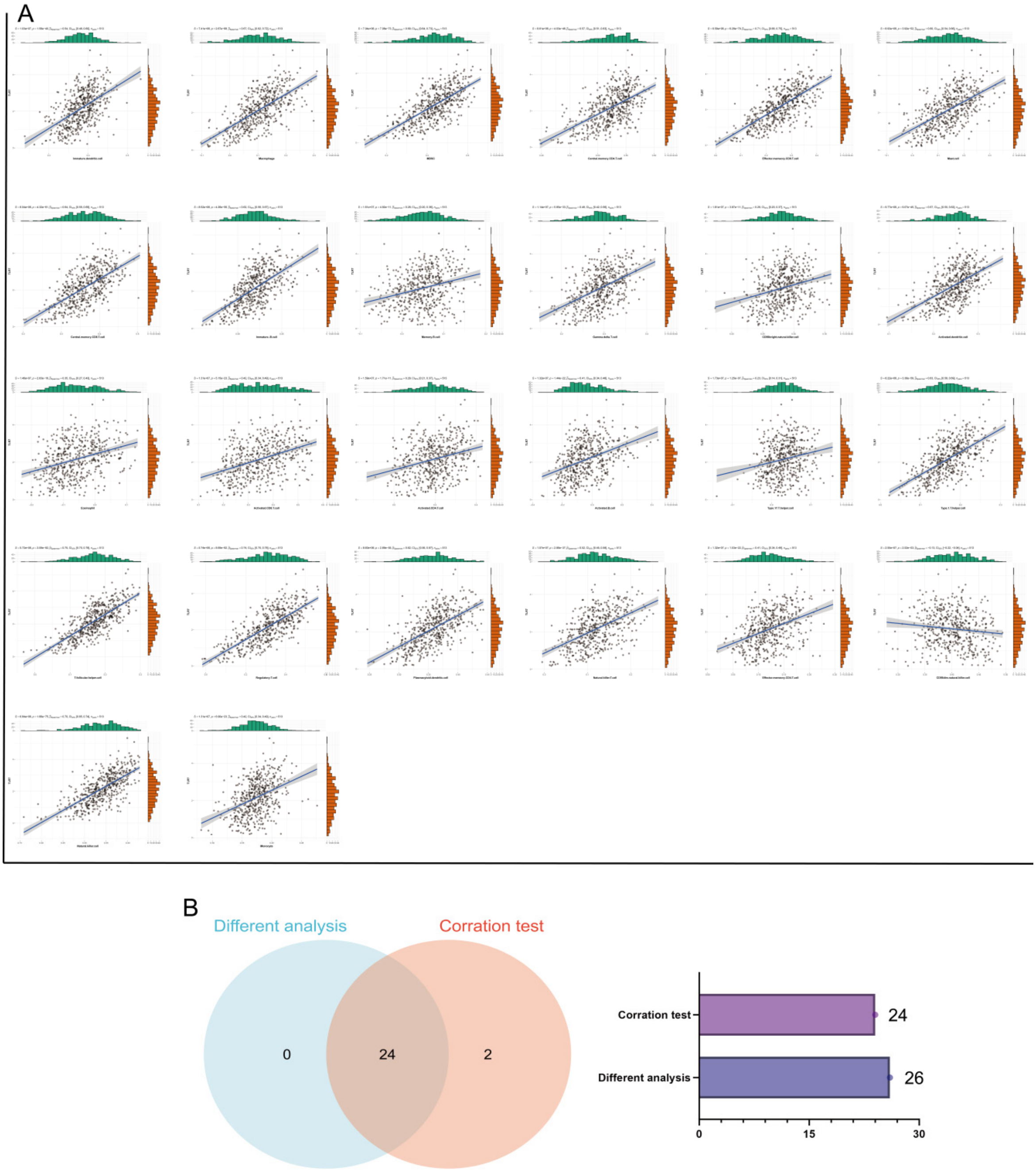

Supplementary Figure S3. Differential analysis of TLR7 expression correlating with tumor-infiltrating immune cells (TIC). (**A**) Scatter plots showing expression levels of 26 TICs correlated with TLR7 expression levels ($p < 0.05$) The blue line in each plot fits a linear model indicating the proportional convergence of immune cells along with TLR7 expression and the Pearson's coefficient was used for correlation tests. (**B**) Venn diagram showing 24 TICs correlated with TLR7 expression, the test of difference and correlation test are shown in the bar chart (Figure 6E) and scatter plot, respectively.

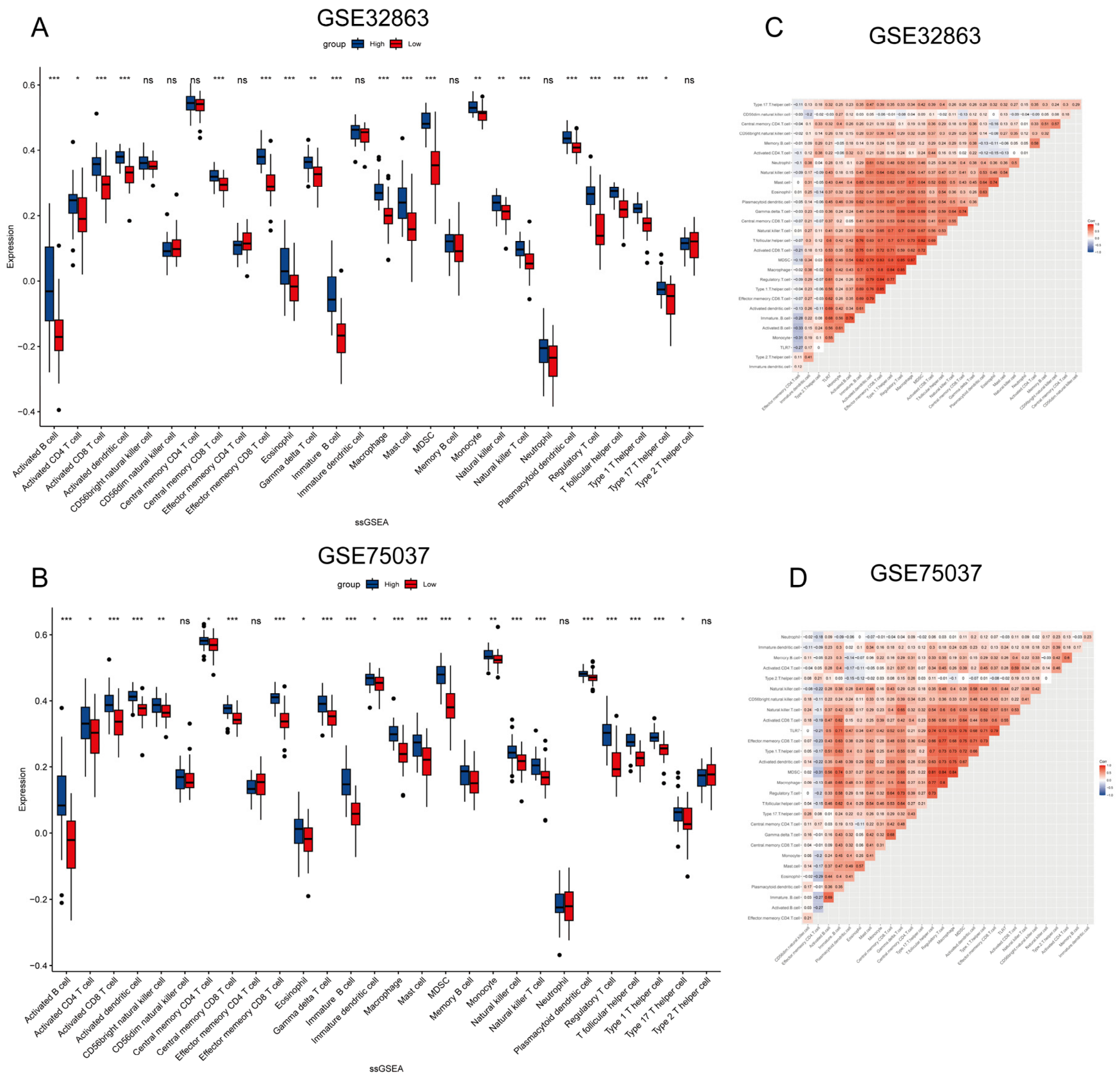

Supplementary Figure S4. TLR7 expression correlates with immune cells infiltrated in LUAD. (**A,B**) Bar graphs showing the distribution of 28 immune cells between LUAD tumor samples with high or low TLR7 expression and significance tests using Wilcoxon rank sum. (**C,D**) Heatmap of the correlation between 28 tumor-infiltrating lymphocytes and TLR7 with the chromaticity of each small colored box representing the corresponding correlation value between the two cells. (***: $p < 0.001$, **: $p < 0.01$, *: $p < 0.05$, ns: not significant).
