# Supplementary material for: TLR7: A Key Prognostic Biomarker and Immunotherapeutic Target in Lung Adenocarcinoma
Source: Biomedicines. 2025 Jan 9;13(1):151. doi: 10.3390/biomedicines13010151 (PMC11761590; doi:10.3390/biomedicines13010151)
Supplement: Supplementary file 1 [file biomedicines-13-00151-s001.zip › Supplementary file S2.pdf]

Supplementary Table S1. Enriched gene sets.

MSigDB Collection	Gene Set Name	NES	NOM p-Adjust	FDR q-Adjust
h.all.v7.0. symbols.gmt TLR7 high expression	HALLMARK_ALLOGRAFT_REJECTION	3.183	1.00E-10	1.58E-10
	HALLMARK_INTERFERON_GAMMA_RESPONSE	3.018	1.00E-10	1.58E-10
	HALLMARK_INFLAMMATORY_RESPONSE	2.927	1.00E-10	1.58E-10
	HALLMARK_IL6_JAK_STAT3_SIGNALING	2.811	1.00E-10	1.58E-10
	HALLMARK_INTERFERON_ALPHA_RESPONSE	2.636	1.00E-10	1.58E-10
	HALLMARK_EPITHELIAL_MESENCHYMAL_TRANSITION	2.275	1.00E-10	1.58E-10
	HALLMARK_IL2_STAT5_SIGNALING	2.194	1.00E-10	1.58E-10
	HALLMARK_COMPLEMENT	2.167	1.00E-10	1.58E-10
	HALLMARK_TNFA_SIGNALING_VIA_NFKB	2.143	1.00E-10	1.58E-10
	HALLMARK_KRAS_SIGNALING_UP	2.061	7.56E-10	9.94E-10
TLR7 low expression	HALLMARK_E2F_TARGETS	-2.145	5.00E-10	1.58E-10
	HALLMARK_OXIDATIVE_PHOSPHORYLATION	-2.072	9.25E-10	2.92E-10
	HALLMARK_MYC_TARGETS_V1	-1.978	6.09E-08	1.92E-08
	HALLMARK_G2M_CHECKPOINT	-1.883	1.35E-06	4.25E-07
	HALLMARK_PANCREAS_BETA_CELLS	-1.976	0	9.99E-05
	HALLMARK_ESTROGEN_RESPONSE_LATE	-1.672	0	0
	HALLMARK_XENOBIOTIC_METABOLISM	-1.640	0	0
	HALLMARK_MYC_TARGETS_V2	-1.827	0	0
	HALLMARK_SPERMATOGENESIS	-1.596	0	0.001
	HALLMARK_MTORC1_SIGNALING	-1.531	0	0.001

Supplementary Table S2. Primer sequence.

	Forward Primer (5′→3′)	Reverse Primer (5′→3′)
TLR7	CACAGCCGTCCCTACTGTTT	TTTTTACACGGCGCACAAGG
Actin	CATGTACGTTGCTATCCAGGC	CTCCTTAATGTCACGCACGAT

Supplementary Table S3. Primary and secondary antibodies and dilution ratio.

Target	Company	Cat. No.	Dilution ratio
TLR7	Abways, China	CY6812	WB 1:1000<br>IHC 1:100
β-actin	Proteintech, China	66009-1-Ig	1:3000
